# A single 36-h water-only fast vastly remodels the plasma lipidome

**DOI:** 10.3389/fcvm.2023.1251122

**Published:** 2023-09-07

**Authors:** Brian V. Hong, Christopher H. Rhodes, Joanne K. Agus, Xinyu Tang, Chenghao Zhu, Jack Jingyuan Zheng, Angela M. Zivkovic

**Affiliations:** Department of Nutrition, University of California, Davis, Davis, CA, United States

**Keywords:** lipidomic, fasting, cardiometabolic health, lipids, free fatty acids

## Abstract

**Background:**

Prolonged fasting, characterized by restricting caloric intake for 24 h or more, has garnered attention as a nutritional approach to improve lifespan and support healthy aging. Previous research from our group showed that a single bout of 36-h water-only fasting in humans resulted in a distinct metabolomic signature in plasma and increased levels of bioactive metabolites, which improved macrophage function and lifespan in *C. elegans*.

**Objective:**

This secondary outcome analysis aimed to investigate changes in the plasma lipidome associated with prolonged fasting and explore any potential links with markers of cardiometabolic health and aging.

**Method:**

We conducted a controlled pilot study with 20 male and female participants (mean age, 27.5 ± 4.4 years; mean BMI, 24.3 ± 3.1 kg/m^2^) in four metabolic states: (1) overnight fasted (baseline), (2) 2-h postprandial fed state (fed), (3) 36-h fasted state (fasted), and (4) 2-h postprandial refed state 12 h after the 36-h fast (refed). Plasma lipidomic profiles were analyzed using liquid chromatography and electrospray ionization mass spectrometry.

**Results:**

Several lipid classes, including lysophosphatidylcholine (LPC), lysophosphatidylethanolamine (LPE), phosphatidylethanolamine, and triacylglycerol were significantly reduced in the 36-h fasted state, while free fatty acids, ceramides, and sphingomyelin were significantly increased compared to overnight fast and fed states (*P* < 0.05). After correction for multiple testing, 245 out of 832 lipid species were significantly altered in the fasted state compared to baseline (*P* < 0.05). Random forest models revealed that several lipid species, such as LPE(18:1), LPC(18:2), and FFA(20:1) were important features in discriminating the fasted state from both the overnight fasted and postprandial state.

**Conclusion:**

Our findings indicate that prolonged fasting vastly remodels the plasma lipidome and markedly alters the concentrations of several lipid species, which may be sensitive biomarkers of prolonged fasting. These changes in lipid metabolism during prolonged fasting have important implications for the management of cardiometabolic health and healthy aging, and warrant further exploration and validation in larger cohorts and different population groups.

## 1. Introduction

Prolonged fasting (PF), defined as caloric restriction for ≥24 h, has emerged as a nutritional strategy for weight loss and improving both lifespan and healthy aging (1, 2). Fasting has been studied as an intervention for various clinical conditions, including chronic inflammation (3) and obesity (4, 5). While these conditions are distinct, they are linked through cardiometabolic health, which underlies most of the chronic diseases of aging (6). For example, improving insulin sensitivity and reducing proinflammatory markers through fasting can have positive effects on cognition (7).

PF exhibits both beneficial and detrimental effects on human metabolism. One mechanism by which PF can beneficially affect health, and in fact has even been found to extend lifespan, is the reduction of oxidative damage, which is thought to be a normal part of the aging process (8). Studies in rats have also shown that PF for 24 h every other day can effectively preserve cognition and attenuate neuroinflammation induced by lipopolysaccharide (9). In addition to its effects on cognition, PF has been shown to have a variety of beneficial effects on physiological processes, including increased autophagy (10), and these benefits on autophagy appear to be more effective when fasted for at least 24–48 h (11). The effects of periodic PF have been documented as safe and beneficial on cardiometabolic and lipid markers in observational and clinical studies (12, 13). Moreover, a single 36-h fast in type 1 diabetic patients displayed a limited risk of dysglycemia (14). This suggests that acute and periodic PF may have potential therapeutic benefits for managing and improving metabolic health. On the other hand, PF can also have deleterious effects on health. For example, the effects of PF on the gut microbiome have been shown across species (15). Studies involving caloric restriction (800 kcal per day) over 8 weeks have exhibited reduced microbial abundance and diversity, a decrease in the production of short-chain fatty acids by gut microbes, and increased susceptibility to pathogens (16). In addition, PF has been shown to reduce lean muscle mass (17). For instance, Dai et al. demonstrated that 6 days of water-only PF reduced lean muscle mass by 9.2%, although in this case lean muscle mass recovered after returning to a normal diet (18).

The effects of PF on the plasma lipidome have gained attention as an area of interest. During fasting periods of 12 to 24 h, there is a metabolic shift from using glucose to utilizing lipid-derived ketone bodies and free fatty acids (FFA) as an energy source in response to caloric deprivation to maintain vital brain and tissue function (10). Lipolysis is upregulated during fasting, where triacylglycerol (TAG) from adipose tissue is hydrolyzed to FFA, which are mobilized into circulation to provide energy (19). Excess fat deposits in adipose tissues, as in the case of obesity, can be detrimental as they may contribute to diseases such as non-alcoholic fatty liver disease (20). Thus, approaches that can deplete the amount of stored fat in overweight and obese individuals are important strategies for improving overall metabolic health. Changes in lipids are critical as lipids constitute a wide range of cellular components, including cell membrane and cell signaling molecules, and disruptions in lipid metabolism have been implicated in several diseases, including metabolic syndrome (21) and Alzheimer's disease (22). Despite the growing interest in the effects of PF and its implications for improving health, little is known about the effects of acute PF on the plasma lipidome. Investigating the plasma lipidome can uncover potential biomarkers of health and point to specific therapeutic strategies to improve cardiometabolic health.

In our previous study, we demonstrated that a single bout of 36-h water-only fasting in humans resulted in a distinct metabolomic signature in plasma, with increased concentrations of bioactive metabolites that enhanced both macrophage function and lifespan in *C. elegans* (23). In this secondary outcome analysis, the aim is to investigate the effects of this intervention on the plasma lipidome. This study addresses the hypothesis that a single bout of 36-h water-only fasting significantly alters the plasma lipidome in expected ways (i.e., increased FFA, decreased TAG) compared to both the postprandial and overnight fasted state, but also that there are changes at the level of total lipid classes as well as individual lipid species, which could be markers of adipose tissue TAG mobilization, and indicators of the overall lipid metabolic effects of PF. We further hypothesized that some of the lipidomic alterations induced by the 36-h fast would extend into the next feeding period, as measured by changes between the fed and refed states.

## 2. Method

### 2.1. Study design

**Figure 1:**
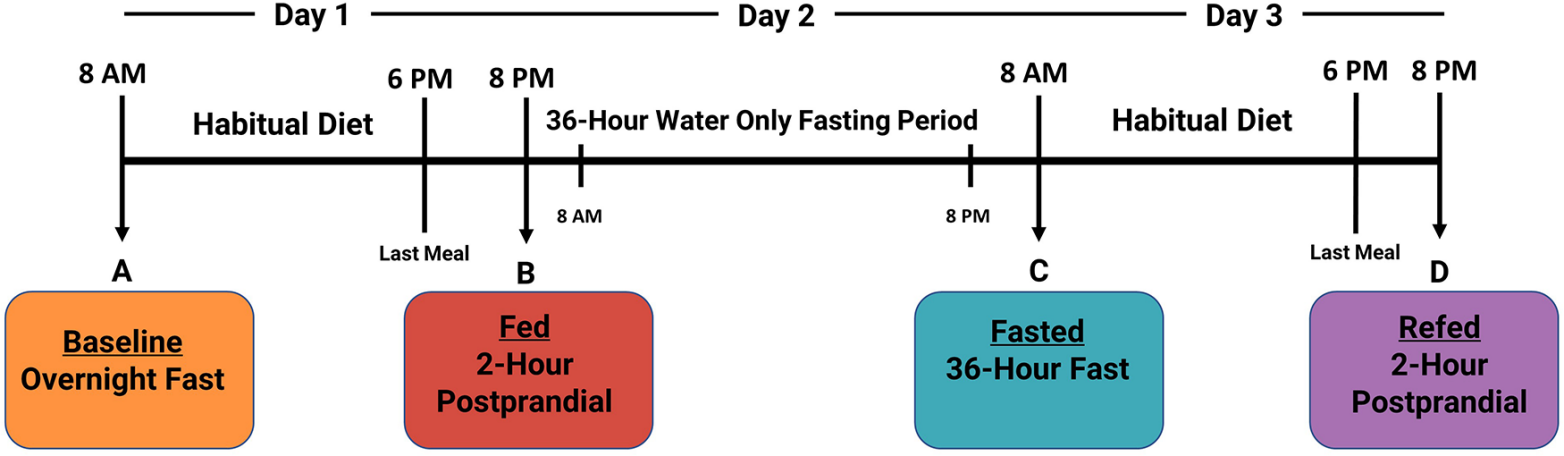
Timeline of 3-day clinical fasting trial. Participants underwent a 3-day clinical fasting trial as follows: (**A**) An overnight fasted blood sample was collected. Afterward, participants return to their normal routine and habitual diet. (**B**) Two hours after eating their last meal on Day 1, a postprandial blood sample was collected, and participants began their 36-h fast (Day 2). (**C**) On Day 3, participants provided a 36-h fasted blood draw and were then instructed to consume the identical diet from Day 1. (**D**) A final 2-h postprandial refed blood sample was collected after the 36-h fasting period.

The complete study design is described in detail elsewhere (23). Plasma was collected from twenty healthy human participants (age: 27.5 ± 4.4; BMI: 24.3 ± 3.1; male: *n* = 10, female: *n* = 10) to measure four distinct nutritional states: (1) overnight fasted (baseline), (2) 2-h postprandial (fed), (3) 36-h fasted (fasted), and (4) an additional 2-h postprandial 12-h after the 36-h fasting period (refed) (Figure 1). Participants were instructed to maintain habitual water consumption and daily routines, except for strenuous exercise. On day 3, before the refed state plasma was taken, participants were instructed to consume the identical diet as on Day 1. The main study protocol was approved by the ethics committees of the University of California, Davis, and was registered on clinicaltrials.gov as NCT03487679.

### 2.2. Plasma collection

Blood samples from all participants were collected using ethylenediaminetetraacetic acid plasma tubes and subjected to centrifugation (1,500 × g) for 10 min at 4°C. Following centrifugation, the samples were partitioned into smaller aliquots. Samples were preserved at −80°C until analysis.

### 2.3. Lipidomic analyses

Lipidomic analysis was performed by Metabolon, Inc using their Complex Lipid Targeted Panel (Morrisville, NC, USA). Briefly, lipids were extracted from plasma in the presence of deuterated internal standards using the butanol:methanol method, as previously described (24). The analysis utilized one or more internal standards for quantifying lipid species, selected from various lipid classes including wax esters, squalene, TAG, diacylglycerols (DAG), FFA, cholesteryl esters (CE), as well as combinations of these classes. During quality control assessment, instrument variability from the lipid panel was determined to be 5%, which was calculated using median relative standard deviation (RSD) for all endogenous metabolites (i.e., non-instrument standards) present in 100% from the quality control pooled sample matrix (25). Identification confidence level is Tier 1 according to the criteria establish by Schymanski et al. (26). The lipid extracts were dried under nitrogen and reconstituted in ammonium acetate dichloromethane:methanol. Samples were analyzed in both positive and negative modes using electrospray ionization on a Shimadzu LC and Sciex Selexon-5500 QTRAP in multiple reaction monitoring mode. For each lipid species, the ratio between the signal intensity of the target compound and its assigned internal standard was taken and multiplied by the concentration of the internal standard. Lipid class concentrations were calculated by summing all molecular species within a class, and fatty acid (FA) composition was determined by the proportion of each class composed of the summation of each FA. In total, 15 lipid classes were measured, which include CE, ceramide (CER), DAG, dihydroceramide (DCER), FFA, hexosylceramide (HCER), lactosylceramide (LCER), lysophosphatidylcholine (LPC), lysophosphatidylethanolamine (LPE), monoacylglycerol (MAG), phosphatidylcholine (PC), phosphatidylethanolamine (PE), phosphatidylinositol (PI), sphingomyelin (SM), and TAG. For lipid species, 832 out of 970 lipid species were included in subsequent analysis after the removal of missing lipids (due to low abundance below the limit of detection) in at least 1 participant at any time point.

### 2.4. Data analysis

Data analysis was performed using R version 4.2.2 (27). Lipid classes and lipid species were analyzed using repeated measures analysis of variance (ANOVA). Prior to analysis, the data were checked for normality using the Shapiro-Wilk test and underwent a log-transformation. If repeated measures ANOVA was significant, *post hoc* analysis was performed using the function pairwise_t_test() from the R package rstatix (28). To address the potential for Type I errors arising from multiple comparisons, the Benjamini-Hochberg method was applied for correction. To calculate log fold changes for volcano plots, linear mixed effects models were carried out on log-transformed lipid species to evaluate differences between groups. Subject ID was included as a random effect, giving each subject an individual intercept using the R package limma. The limma function lmFit() and eBayes() were then utilized to calculate moderated t-statistics for identifying differentially expressed lipids, followed by adjusting for multiple comparisons using the Benjamini Hochberg method (29). Each model was generated pairwise and encompassed two comparisons: baseline vs. fasted and fed vs. refed states. Lipid species that were missing in one participant at any time point were removed from the analysis. Dimension reduction was performed using principal component analysis (PCA). A *P* < 0.05 was considered significant.

Random Forest (RF) models with cross-validation (number of partitions = 5) were used as an unbiased supervised machine learning approach for classification using the Tidymodels package (30). This method is based on the ensemble of decision trees (31) and is used to make predictions and estimate class prediction accuracy. The advantages of RF include its robustness and unbiased approaches to handling skewed and multimodal data, as well as reducing overfitting. To generate a subset of random data for training, we applied random feature selection methods. This subset was then used to evaluate how well the RF model could predict sample class in the test dataset.

Saturation level of FA was determined by categorizing each lipid class based on the number of double bonds present in their respective FA. FA were classified as saturated, monounsaturated, or polyunsaturated depending on whether they had 0, 1, or 2 or more double bonds, respectively. FA species that were not present in one of the participants at any time point were excluded from the analysis. Total FA concentration for each lipid class was calculated by summing the concentrations of FA within each saturation level. The data was then log-transformed for linear modeling using the R package limma (29) to calculate log fold changes. Multiple testing was corrected using the Benjamini Hochberg method.

## 3. Results

### 3.1. Lipidomic alterations during a 36-h prolonged fast in healthy participants

**Figure 2:**
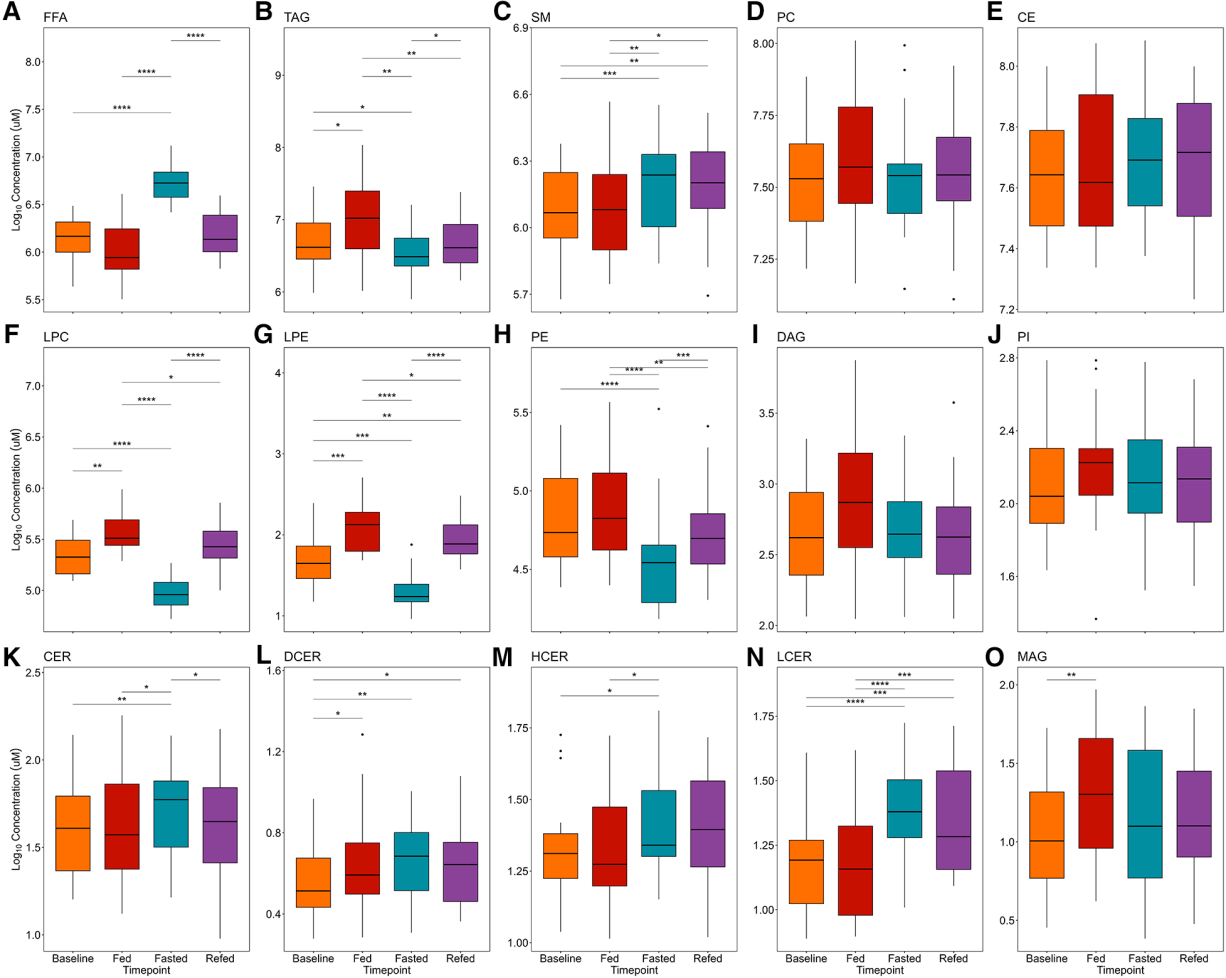
Effects of prolonged fasting on plasma lipid classes. Fifteen lipid classes were measured in four nutritional states: (1) overnight fast (baseline), (2) 2-h postprandial (fed), (3) 36-h water-only fast (fasted), and (4) 2-h postprandial after a 36-h water-only fast (refed). Boxplots show plasma concentrations of the following lipid classes: (**A**) FFA, free fatty acid; (**B**) TAG, triacylglycerol; (**C**) SM, sphingomyelin; (**D**) PC, phosphatidylcholine; (**E**) CE, cholesteryl ester; (**F**) LPC, lysophosphatidylcholine; (**G**) LPE, lysophosphatidylethanolamine; (**H**) PE, phosphatidylethanolamine; (**I**) DAG, diacylglycerol; (**J**) PI, phosphatidylinositol; (**K**) CER, ceramide; (**L**) DCER, dihydroceramide; (**M**) HCER, hexosylceramide; (**N**) LCER, lactosylceramide; (**O**) MAG, monoacylglycerol. Significance between states was determined by repeated measures ANOVA followed by *post hoc* pairwise *t*-tests. *P*-values were adjusted using the Benjamini Hochberg method for all pairwise comparisons. *P < 0.05, **P < 0.01, ***P < 0.001, ****P < 0.0001.

Participant baseline characteristics are presented in Table 1. Detailed participant characteristics across the 4 nutritional states are presented elsewhere (23). FFA, TAG, SM, PC, and CE lipid classes were the most abundant, while CER and their analogs DCER, HCER, and LCER, as well as MAG were the least abundant (Figure 2). The repeated measures ANOVA results are presented in Supplementary Table S1 and the median and 95% confidence interval for the lipid classes for all nutritional states are presented in Supplementary Table S2. As expected, FFA were significantly increased in the 36-h fasted state compared to the other 3 states (*P* < 0.001 for all, Figure 2A), while TAG was significantly decreased compared to the overnight fasted (*P* < 0.05), fed (*P* < 0.01), and refed state (*P* < 0.05) (Figure 2B). SM was significantly increased in the fasted state compared to baseline (*P* < 0.001) and fed state (*P* < 0.01) and remained significantly elevated in the refed state compared to baseline (*P* < 0.01) and the fed state (*P* < 0.05) (Figure 2C). PC [F(3,57) = 2.02] and CE [F(3,57) = 1.24] were not significantly different across the four states by repeated measures ANOVA (*P* > 0.05 for both, Figures 2D,E). LPC and LPE were significantly lower in the 36-h fasted state compared to the overnight fasted, fed and refed states (*P* < 0.001 for all, Figures 2F,G), while LPC and LPE remained significantly lower in the refed state compared to the fed state (*P* < 0.05). PE was significantly decreased in the fasted state compared to the baseline (*P* < 0.0001), fed (*P* < 0.0001), and refed states (*P* < 0.001, Figure 2H) and remained significantly decreased in the refed state compared to the fed state (*P* < 0.01, Figure 2H). DAG [F(3,57) = 2.57] and PI [F(3,57) = 0.90] were not significantly different across the four states by repeated measures ANOVA (*P* > 0.05 for both, Figures 2I,J). CER was significantly increased in the 36-h fasted state compared to the overnight fast (*P* < 0.01, Figure 2K), as were the analogs DCER (*P* < 0.01, Figure 2L), HCER (*P* < 0.05, Figure 2M), and LCER (*P* < 0.0001, Figure 2N). DCER was significantly increased in the fed state compared to overnight fast (*P* < 0.05. Figure 2L). HCER was significantly higher in the 36-h fasted state compared to fed (*P* < 0.05, Figure 2M). DCER remained significantly elevated in the refed state compared to baseline (*P* < 0.05), while LCER remained significantly elevated in the refed state compared to both the baseline (*P* < 0.001) and fed states (*P* < 0.001). MAG was significantly increased in the fed state compared to baseline (*P* < 0.01, Figure 2O).

**Table 1:** Participant baseline characteristics.

Participant characteristic	Baseline
Sample size (*N*)	20
Age (year)	27.5 ± 4.4
Height (cm)	170.7 ± 9.8
Weight (kg)	71.3 ± 13.4
BMI (kg/m^2^)	24.3 ± 3.1
Waist circumference (cm)	78.8 ± 8.9
Systolic blood pressure (mmHg)	113.7 ± 9.0
Diastolic blood pressure (mmHg)	71.6 ± 5.5
HDL cholesterol (mg/dl)	66.1 ± 16.8
LDL cholesterol (mg/dl)	77.5 ± 27.0
Triglyceride (mg/dl)	94.4 ± 31.6
Total Cholesterol (mg/dl)	163.1 ± 36.3

### 3.2. Prolonged fasting induces distinct changes in plasma lipidome profiles

**Figure 3:**
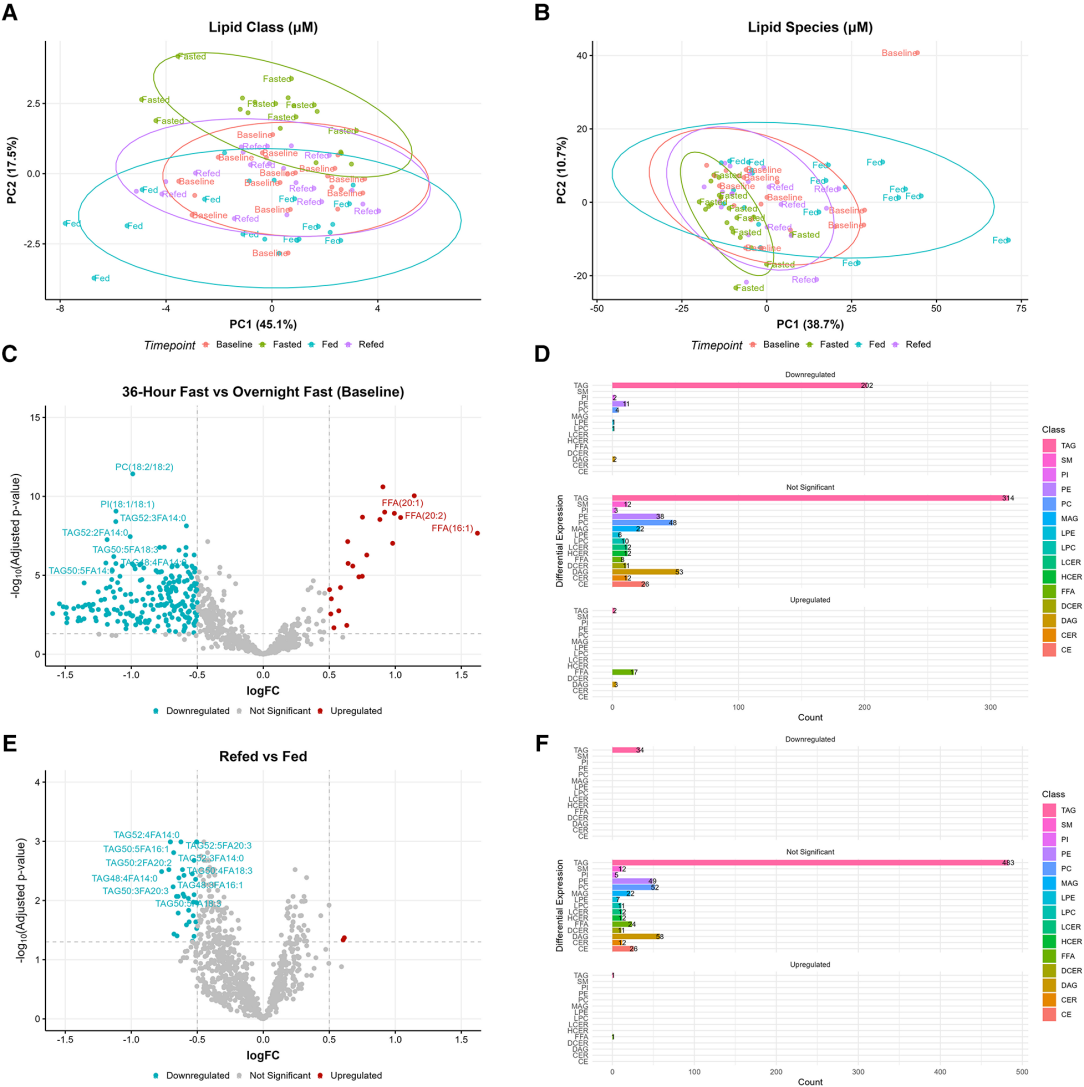
Alterations in lipid species during prolonged fasting. Principal component analysis (PCA) revealed clustering of the 36-h fasted state in (**A**) lipid classes and (**B**) lipid species. (**C**) Volcano plot comparing the 36-h fasted state vs. overnight fasted baseline, where higher logFC values indicate higher concentrations in the 36-h fasted state. (**D**) Barplot showing the number of differentially expressed lipid species between the 36-h fasted state and baseline. (**E**) Volcano plot comparing the refed state vs. fed state, where higher logFC values indicate higher concentrations in the refed state. (**F**) Barplot showing the number of differentially expressed lipid species between the refed state and fed state. Both volcano and barplots are considered significant with an absolute logFC > 0.5 and adjusted *P*-value < 0.05.

PCA analysis was conducted to explore the distribution of lipids across the four nutritional states (baseline, fed, 36-h fasted, and refed) (Figures 3A,B). PCA revealed clustering of participants in the 36-h fasted state, indicating a lipid profile characteristic of this state across participants (Figure 3B). In contrast, the lipidomic profiles of the baseline, fed, and refed states did not separate from each other by multivariate analysis (Figures 3A,B). Analyses were then focused on two comparisons: (1) the overnight fasted baseline state vs. the 36-h fasted state and (2) the fed vs. the refed state. A list of lipid species, along with their log fold changes and adjusted *P*-values, between the fasted vs. baseline and the refed vs. fed states can be found in Supplementary Table S3 and Supplementary Table S4, respectively. We identified 832 lipid species using an absolute log fold change cutoff of 0.5 and adjusted *P*-value < 0.05. Lipid species were ranked by both adjusted *P*-values and absolute logFC and the top 10 ranked lipid species are shown. In the 36-h fasted state 22 lipid species were significantly upregulated, while 223 lipid species were significantly downregulated compared to the baseline overnight fasted state (Figure 3C). Among the downregulated lipid species, there were 202 TAGs, 2 PI species including PI(18:0/18:1) and PI(18:1/18:1), 11 PEs, 4 PCs, LPE(18:1), LPC(18:2), and 2 DAGs (Figure 3D). Of the lipid species upregulated, 2 were TAGs, 17 were FFAs, and 3 were DAGs. When comparing the refed vs. the fed state, 34 lipid species were significantly downregulated in the refed state, while only 2 lipid species were significantly upregulated (Figure 3E). All 34 of the downregulated species were TAGs, whereas the upregulated lipid species included 1 TAG and FFA(16:1) (Figure 3F). Out of the top 10 lipid species significantly altered, myristic acid (FA14:0) containing TAG such as TAG52:3FA14:0 and TAG48:4FA14:0 remained significantly decreased in the refed state in addition to alpha-linolenic acid containing TAG such as TAG50:5FA18:3.

Analysis of the 518 TAG species, spanning a range of 36 to 60 carbon atoms and 0 to 12 double bonds, revealed distinct patterns of change (Supplementary Figure S1). TAG species clustered within the range of 40 to 58 carbon atoms and ≤8 double bonds, primarily associated with saturated FA (SFA) (FA:12:0, FA14:0, FA15:0, FA16:0, FA17:0, FA18:0) and monosaturated FA (MUFA) (FA18:1 and FA20:1) decreased. Similarly, TAG species containing linoleic (FA18:2) and alpha-linolenic (FA18:3) also showed a decrease. TAG species with FA containing 20 carbon atoms and ≤3 doubles bonds were predominately reduced within the range of 50–56 carbon atoms and ≤3 doubles bonds. In contrast, TAG species linked with FA14:1 or FA with more than 20 carbon atoms and ≥4 doubles bonds (FA22:1, FA20:4, FA22:4, FA20:5, FA22:5, FA22:6), showed minimal changes. Comparing the refed vs. fed states, TAG species with carbon atom ranges between 40 and 56 and ≤7 double bonds, linked with FA14:0, FA16:1, FA20:1, FA20:2, FA18:3, or FA20:3, remained low (Supplementary Figure S2). In terms of LPC species, those with 15 to 20 carbon atoms and ≤3 double bonds decreased in the 36-h fasted state compared to baseline (Supplementary Figure S3A). Only LPC(18:0) decreased in the refed state but with a low log fold change (−0.19) compared to the fed state (Supplementary Figure S3B). Regarding LPE species, those with 16 to 20 carbon atoms and ≤3 double bonds decreased during the 36-h fasted state compared to baseline, while no changes in LPE species were observed between the refed and fed state (Supplementary Figures S4A,B).

### 3.3. Random forest analysis reveals key lipid species in differentiating nutritional states

**Figure 4:**
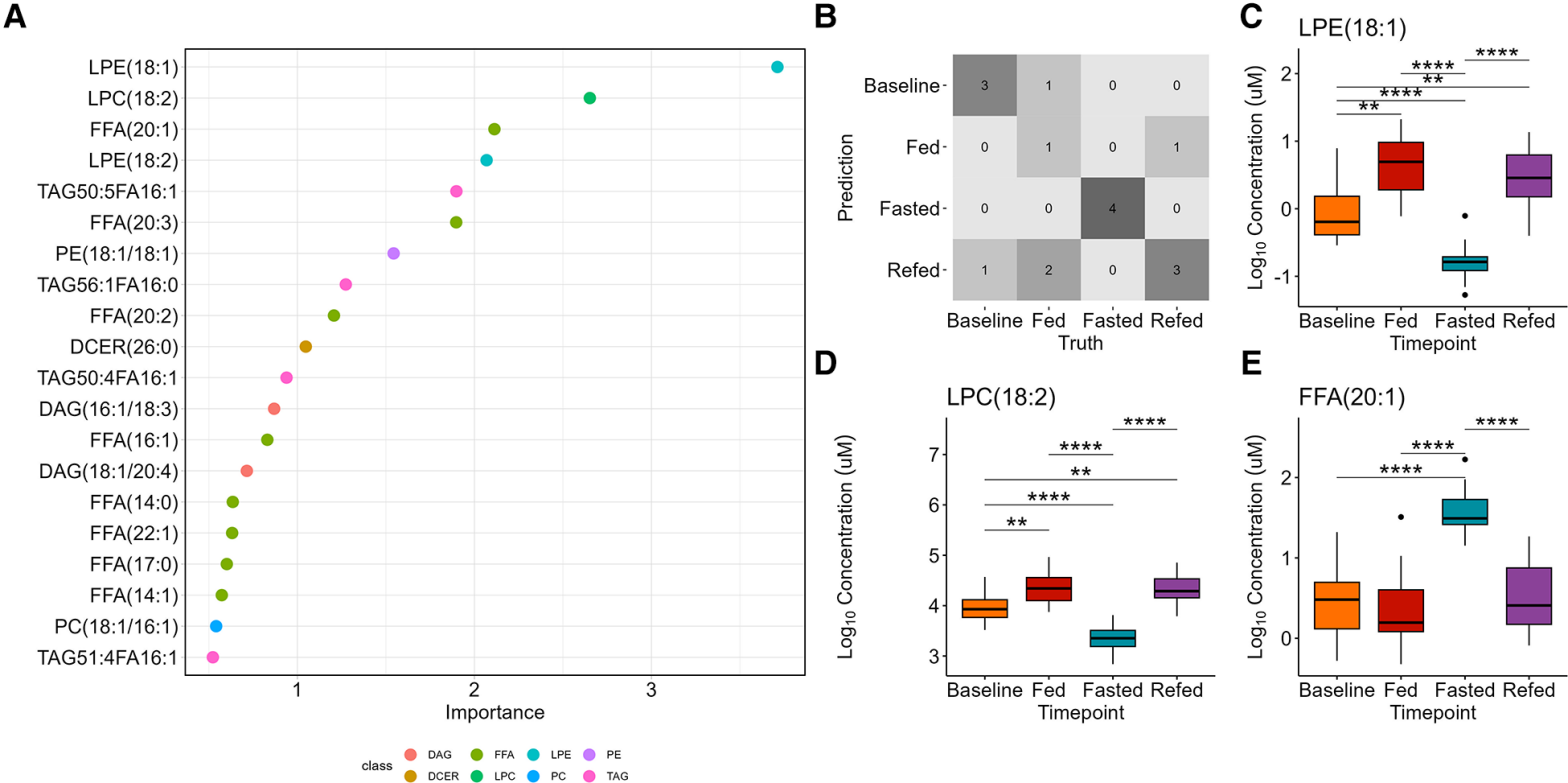
Random forest classification of the four nutritional states. (**A**) The variable importance plot shows the mean decrease in impurity for each variable, indicating their importance in determining model accuracy. (**B**) The confusion matrix displays the prediction accuracy of the model, showing the number of correctly predicted samples for each class and the misclassification rates. The accuracy of the multiclass model is 69%. The top three important variables are (**C**) LPE(18:1), which is identified as one of the most important variables in determining the model accuracy, followed by (**D**) LPC(18:2), and E) FFA(20:1).

Random forest analysis was used as a multiclass classification model to differentiate between the four nutritional states (Figure 4A). The model assigns variable importance scores to the predictors (lipid species) based on their contribution to the model's accuracy, using the mean decrease in impurity method. LPE(18:1) was the strongest predictor with a variable score of 3.7, followed by LPC(18:2) (score = 2.7) and FFA(20:1) (score = 2.1). Supplementary Table S5 presents the variable importance scores for the remaining predictors. A confusion matrix indicates the prediction accuracy (69%) of the model (Figure 4B). LPE(18:1) and LPC(18:2) were significantly decreased whereas FFA(20:1) was significantly increased in the 36-h fasted state compared to the other three states (*P* < 0.0001 for all, Figures 4C–E).

### 3.4. Fatty acid saturation altered by prolonged fasting

**Figure 5:**
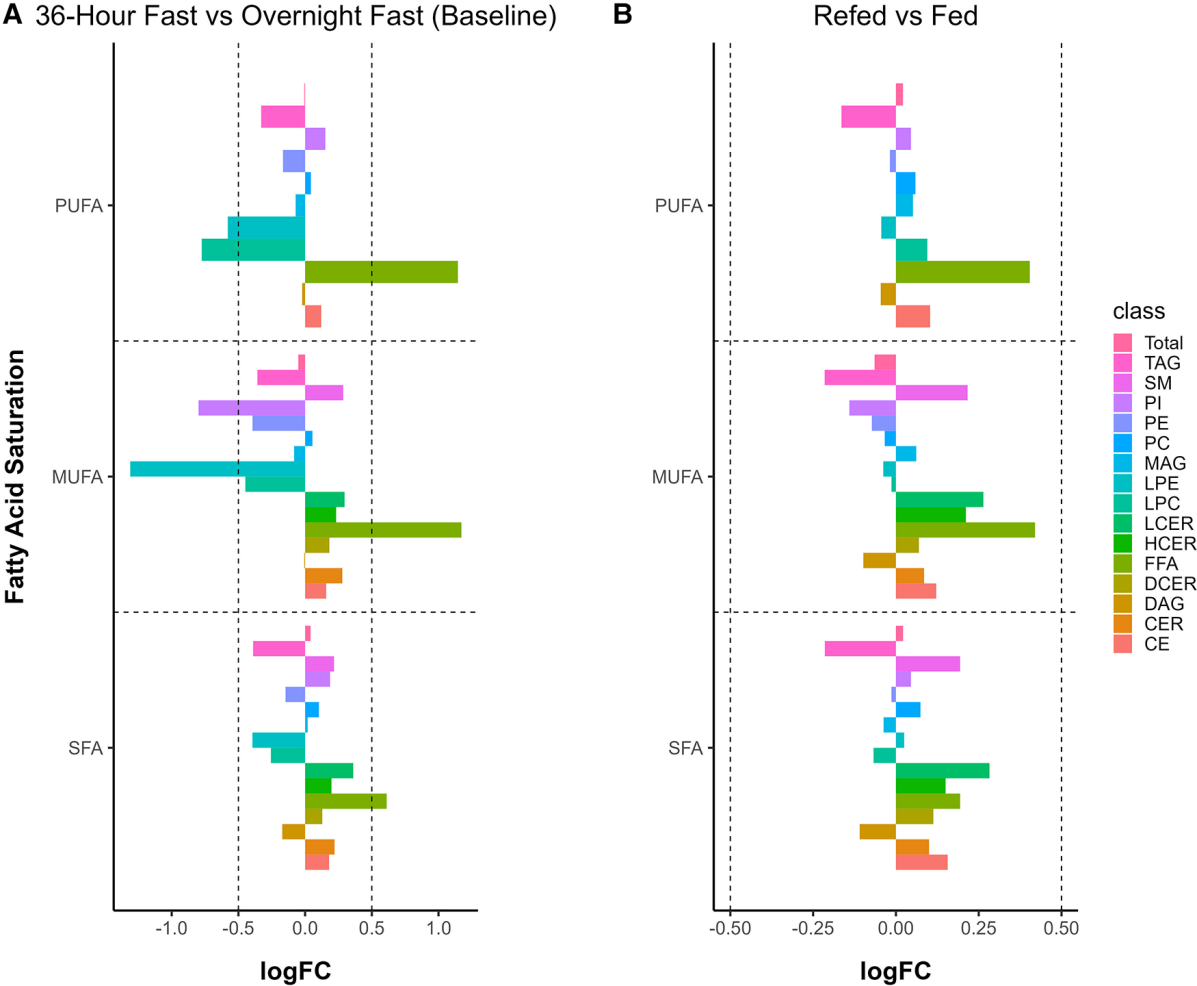
Alteration in fatty acid saturation by a 36-h fast. (**A**) Monounsaturated fatty acids (MUFA) and polyunsaturated fatty acids (PUFA) decreased in the 36-h fast, whereas free fatty acids (FFA) increased at all three levels of saturation. (**B**) Saturation levels between refed and fed states. Changes in saturation are considered significant with both an absolute logFC > 0.5 and an adjusted *P*-value < 0.05.

FA saturation level was compared between the 36-h fasted state vs. baseline and the refed vs. fed states using an absolute log fold change cutoff of 0.5 and adjusted *P*-value < 0.05. In the comparison of the 36-h fasted state vs. baseline, MUFA LPE and PI were significantly reduced (*P* < 0.001 for both), as well as polyunsaturated acids (PUFA) LPE and LPC (*P* < 0.001 for both, Figure 5A). In contrast, FFA SFA, MUFA, and PUFA were significantly increased (*P* < 0.001 for all, Figure 5A). For the refed vs. fed states, we did not observe any significant differences in saturation levels across all lipid classes when using an absolute log fold change cutoff of 0.5 (Figure 5B).

## 4. Discussion

PF has been demonstrated to be an effective strategy for promoting anti-inflammatory meditators and improving lifespan in animal models (1). Previously, we showed that that plasma metabolome is differentially altered by a 36-h fast, promoting metabolites such as spermidine, 1-methylnicotinamide, palmitoylethanolamide, and oleoylethanolamide, which in combination improved lifespan in *C. elegans* (23). In this secondary outcome analysis, we comprehensively examine the acute effects of PF on the plasma lipidome. Using a lipidomic platform capable of quantitative compositional analysis and complete speciation data, we observed significant reductions in total LPC and LPE levels during the 36-h fasted state, specifically marked decreases in LPE(18:1) and LPC(18:2). Additionally, our analysis of TAG revealed a pattern of decreased TAG species, particularly those linked with SFA and MUFA, which remained decreased in the refed state, providing unprecedented detail regarding the specific TAG carbon composition and their associated fatty acids. These findings provide novel insights into the metabolic adaptations associated with PF, highlighting the intricate relationship between fasting, the plasma lipidome, and metabolic processes, with potential implications for cardiometabolic health and aging.

As expected, FFA were markedly increased in the 36-h fasted state, consistent with a metabolic shift to the mobilization and utilization of stored lipids (32). A previous study showed plasma FFA, measured by an enzymatic colorimetric assay, were significantly elevated 2.8-fold from a 24-h fast compared to an overnight fast in healthy young adults (33), while in this study a 1.8-fold increase in circulating FFA was observed after a 36-h fast. These differences are likely due to the difference in methods used to quantify FFA. Many different FFA species were increased in the fasted state, consistent with net mobilization of stored fat from the adipose. FFA(16:1) or palmitoleic acid, was the lipid species with the highest log-fold change increase from the baseline overnight fasted state to the 36-h fasted state, and it also remained elevated in the refed state vs. the fed state. Palmitoleic acid is a primary product of the *de novo* lipogenesis pathway (34), and has been hypothesized to be an adipose-derived hormone or lipokine associated with insulin sensitivity in overweight individuals (35). Notably, palmitoleic acid is enriched in visceral adipose tissue compared to subcutaneous adipose tissue, as observed from FA analysis of biopsy samples from 75 human participants (36), suggesting that the higher increase in palmitoleic acid relative to other FFA could be indicative of preferential mobilization of fat specifically from the visceral adipose tissue in the 36-h fasted state. This has important implications for the management of metabolic health since increases specifically in visceral fat are associated with inflammation and metabolic dysfunction, whereas increased subcutaneous fat is less metabolically active (37, 38).

Also as expected, TAG was decreased in the 36-h fasted state, consistent with increased TAG metabolism and utilization (19). Hypertriglyceridemia is one of the criteria for diagnosing metabolic syndrome (39), and increased circulating TAG is associated with an increased risk of cardiovascular disease (40). Importantly, the concentrations of TAG in the refed state remained significantly decreased compared to the fed state, suggesting a lasting effect of the 36-h fast on plasma lipids. Our analysis revealed that TAG species with carbon atoms ranging from 40 to 58 carbon atoms and ≤8 double bonds were predominately decreased during the 36-h fast, with specific changes dependent on the FA linkage. SFA and MUFA linked to TAG species showed a decrease, except for TAG species containing FA14:0, FA22:1, or FA with more than 20 carbon atoms and ≥4 doubles bonds. In the PREDIMED trial (Prevención con Dieta Mediterránea), TAG species ≤56 carbon atoms and ≤3 double bonds were associated with increased incidence of type 2 diabetes, while odd-chain TAGs adjusted for total TAG showed an inverse association; however, FA linkage to TAG was not measured (41). Similarly, the Bruneck Study showed several TAG with 50 to 56 carbon atoms and ≤4 double bonds were positively associated with incident of cardiovascular disease, yet FA linkage to TAG was not measured (42). These findings underscore the significance of considering FA linkage when studying TAG species, as it provides valuable insights into their potential associations with cardiometabolic health.

Both LPC and LPE are involved in inflammation, signal transduction, and metabolism (43). LPC and LPE are derived from the hydrolysis of PC and PE, respectively, both of which are important in regulating lipid metabolism associated with health and disease (44). LPC is the most abundant lysoglycerophospholipid in plasma with a reported range of 200–300 μM (43), similar to the range in our cohort..

In our cohort, we observed significant decreases in the levels of LPE(18:1) and LPC(18:2) during the 36-h fasted state compared to both the overnight fasted and fed states. This finding is consistent with a study by Steinhauser et al., which demonstrated that several plasma LPC and LPE lipid species, including LPE(18:1) and LPC(18:2), were decreased during periods of starvation lasting between 1 and 10 days compared to an 8-h overnight fast (45). Furthermore, plasma LPE(18:1) has been found to be elevated in Alzheimer's disease and has been suggested to be a potential early biomarker for the disease (46). Besides their role in cardiovascular disease and cognition, plasma LPC levels are elevated in patients with insulin-dependent diabetes (47) and ovarian cancer (48), while both LPC and LPE are elevated in renal disease patients (49). Moreover, a study utilizing shotgun lipidomics revealed increased levels of several plasma lipid classes, including both total LPE and PE, 3-h postprandial after a high-fat meal challenge compared to the levels observed after an overnight fast, whereas changes in total LPC was not reported (50). Overall, the decreased concentrations of LPC and LPE in the 36 h fasted state likely reflect changes in lipid metabolism, and the effects of PF on these markers might have health implications.

Sphingolipids, including SM, CER, and their analogs DCER, HCER, and LCER are a family of lipids involved in cell recognition and signal transduction (51, 52). These lipids were found to be increased in a 36-h fasted state, with SM being one of the most abundant lipid classes in plasma. Soeters et al. observed that a short-term 62-h fast, as compared to a 14-h fast, in healthy lean men led to a rise in intramuscular ceramide concentrations and induced peripheral insulin resistance, which may serve as a protective mechanism against hypoglycemia (53). In a separate study involving healthy young male participants, sphingomyelin and ceramides were found to be increased in muscle after moderate training (54). The observed increase in plasma ceramides and sphingomyelins during a 36-h fast could be attributed to the body's adaptation to the altered lipid metabolism and energy demands associated with the fasted state. In contrast, bioactive sphingolipids, including ceramides, have been shown to be elevated in neurodegenerative diseases, such as Alzheimer's disease and Parkinson's disease (55–57). For example, several species of plasma ceramides, including CER(16:0), CER(18:0), CER(20:0), CER(22:0), and CER(24:1), were found to be positively correlated with Parkinson's disease and cognitive impairment, but total ceramide was not measured (56). Similarly, in Alzheimer's disease patients, plasma ceramides CER(16:0), CER(18:0), and CER(24:1), were increased compared to normal elderly control subjects, but total ceramide was not measured (57). In our study, CER(24:0), DCER(24:0). and LCER(16:0) were found to be significantly increased after a 36-h fast compared to baseline after multiple testing corrections, but the log fold changes were small (logFC < 0.2, Supplementary Table S3). The alteration of sphingolipid metabolism has been linked to mechanisms that affect cell survival and protein aggregation, such as amyloid beta, as reviewed elsewhere (58). However, the implications of short-term increase in ceramides and sphingomyelins during fasting vs. chronically elevated levels in neurodegenerative diseases remain unclear. More research is needed to understand the links between fasting-induced lipid changes and sphingolipid dysfunction in age-related cognitive decline.

We found that total PI concentration was not significantly different across the four nutritional states, while total MUFA PI species significantly decreased in the 36-h fast compared to the overnight fast. PI serves as a signaling molecule in the cell membrane involved in insulin signaling and glucose uptake (59). Fasting increases fatty oxidation and lipolysis which may change lipid composition within the cell membrane (19). These changes during fasting may be more specific to PI MUFA metabolism. In addition, we found that plasma PI(18:1/18:1) was decreased in the 36-h fast compared to baseline. PI(18:1/18:1) is a lipokine produced by stearoyl-CoA desaturase 1 and has been shown in fibroblast to control stress response and decrease cell death (60). This finding suggests that the observed decrease in plasma PI(18:1/18:1) during the 36-h fast might be linked to changes in cellular stress response and survival mechanisms. Further research is needed to understand the role of PI during fasting and its potential implications for cardiometabolic health and aging, as well as the possible role of PI(18:1/18:1) as a sensitive biomarker of prolonged fasting.

A major strength of this study is the targeted quantitative analysis of the plasma lipidome, offering a precise and dependable evaluation of overall lipid alterations during PF. Furthermore, the same participants were measured at four different time points, which strengthens the internal validity of the study by reducing inter-individual variability. However, certain limitations should be considered. The small sample size limits the generalizability of our findings, necessitating further studies with larger, more diverse cohorts for validation. Additionally, the participants were healthy young adults recruited from the Davis, CA area, which may not fully represent the general population, further emphasizing the need for broader, more diverse samples in future investigations. Lastly, while our study reveals complex lipidomic changes during acute PF in a cohort of healthy young individuals, further research is needed to explore the underlying mechanisms of PF and their health implications more thoroughly, particularly in older or diseased populations. Future research could explore the long-term effects of repeated PF cycles on lipid metabolism and assess the potential benefits for individuals at risk of cardiometabolic diseases. Investigating the connection between PF and the sustained decrease in TAG, PE, and lysophospholipid levels with overall health outcomes would be particularly beneficial for individuals with metabolic or dyslipidemic disorders.

In summary, we used targeted quantitative analysis to investigate the acute effects of PF on the plasma lipidome. Our findings revealed marked changes in various lipid classes, such as FFA, TAG, LPC, LPE, PE, and sphingolipids during a 36-h fast. These alterations in lipid metabolism could be attributed to the body's adaptation to the fasted state, with potential implications for cardiometabolic health and aging. This secondary outcome analysis contributes valuable insights into the complex lipidomic changes occurring during PF and establishes a foundation for future research to elucidate the underlying mechanisms and health implications of these lipid alterations.

## Data Availability

The original contributions presented in the study are included in the article/**Supplementary Material**, further inquiries can be directed to the corresponding author.
