# Supplementary material for: A single 36-h water-only fast vastly remodels the plasma lipidome
Source: Front Cardiovasc Med. 2023 Sep 7;10:1251122. doi: 10.3389/fcvm.2023.1251122 (PMC10513913; doi:10.3389/fcvm.2023.1251122)
Supplement: Supplementary file 6 [file Datasheet1.docx]

**A single 36-hour water-only fast vastly remodels the plasma lipidome**

**Supplementary Figures S1-S4**

Brian V. Hong^1^, Christopher H. Rhodes^1^, Joanne K. Agus^1^, Xinyu Tang^1^, Chenghao Zhu^1^, Jack Jingyuan Zheng^1^, Angela M. Zivkovic^1*^

^1^Department of Nutrition, University of California, Davis, Davis, CA, USA


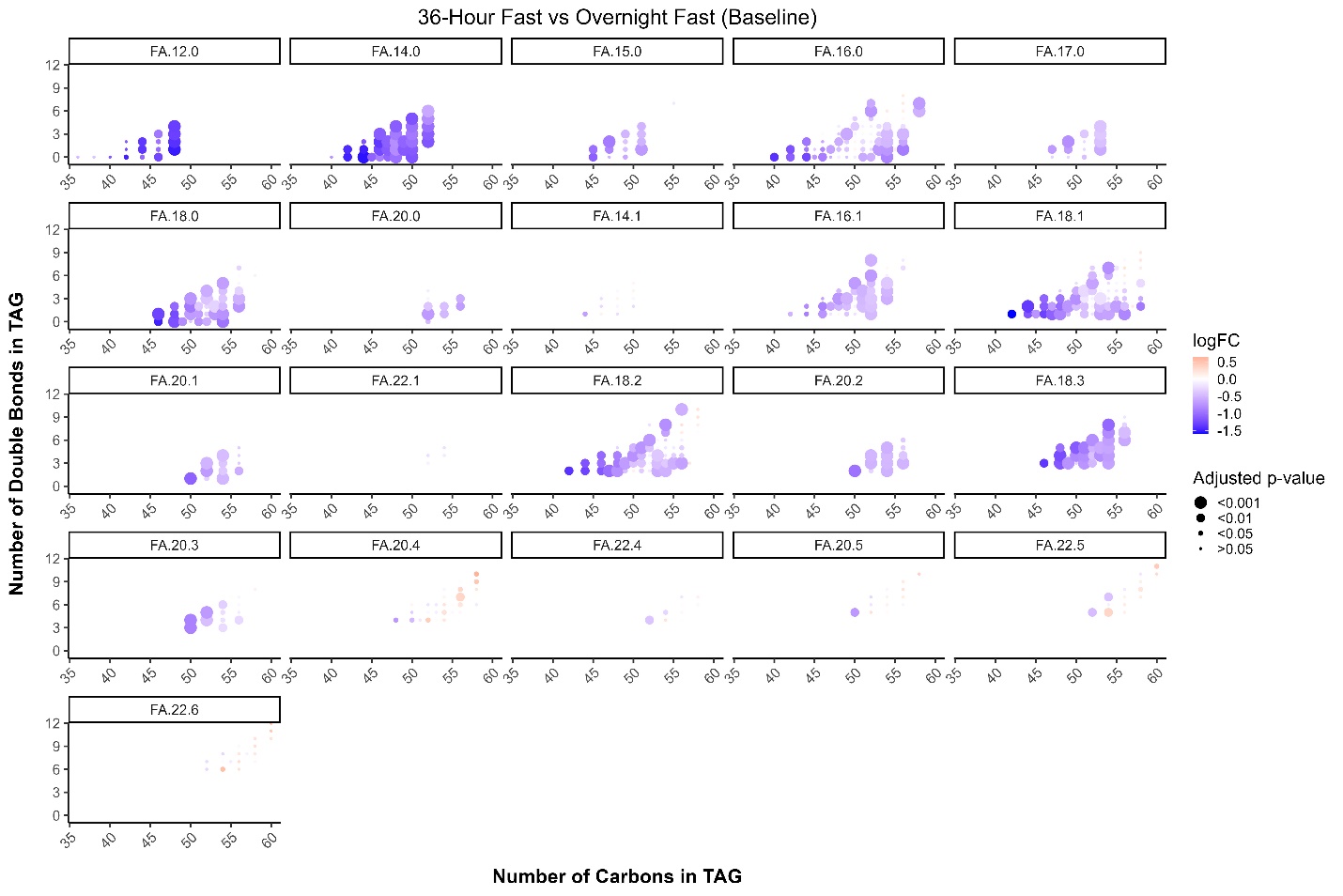


**Supplementary Figure S1.** Triacylglycerol (TAG) species comparing the 36-hour fasted state vs overnight fasted baseline, where higher logFC values indicate higher concentrations in the 36-hour fasted state. Data is stratified by fatty acid (FA) carbon number and saturation level.


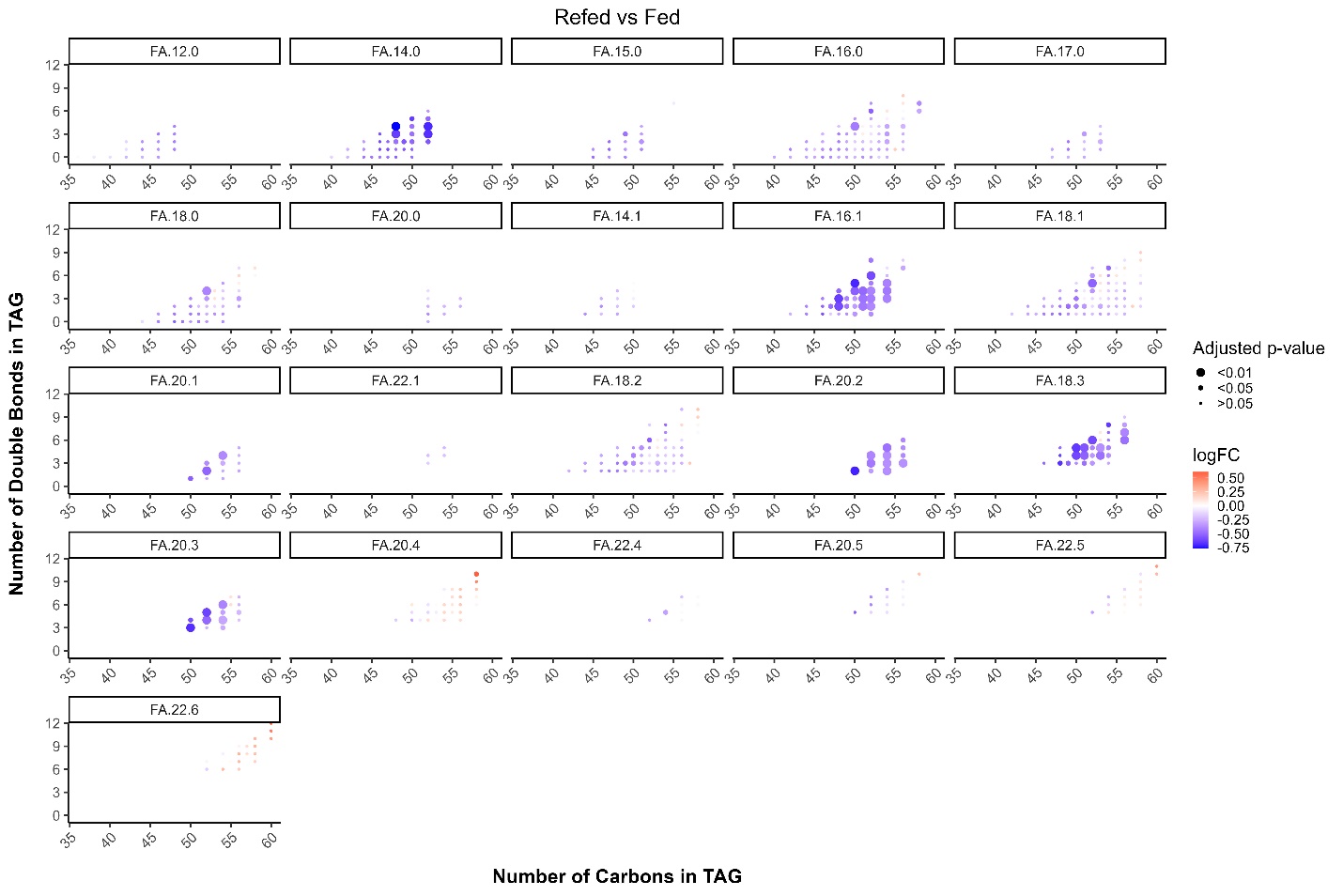


**Supplementary Figure S2.** Triacylglycerol (TAG) species comparing the refed state vs fed state, where higher logFC values indicate higher concentrations in the refed state. Data is stratified by fatty acid (FA) carbon number and saturation level.


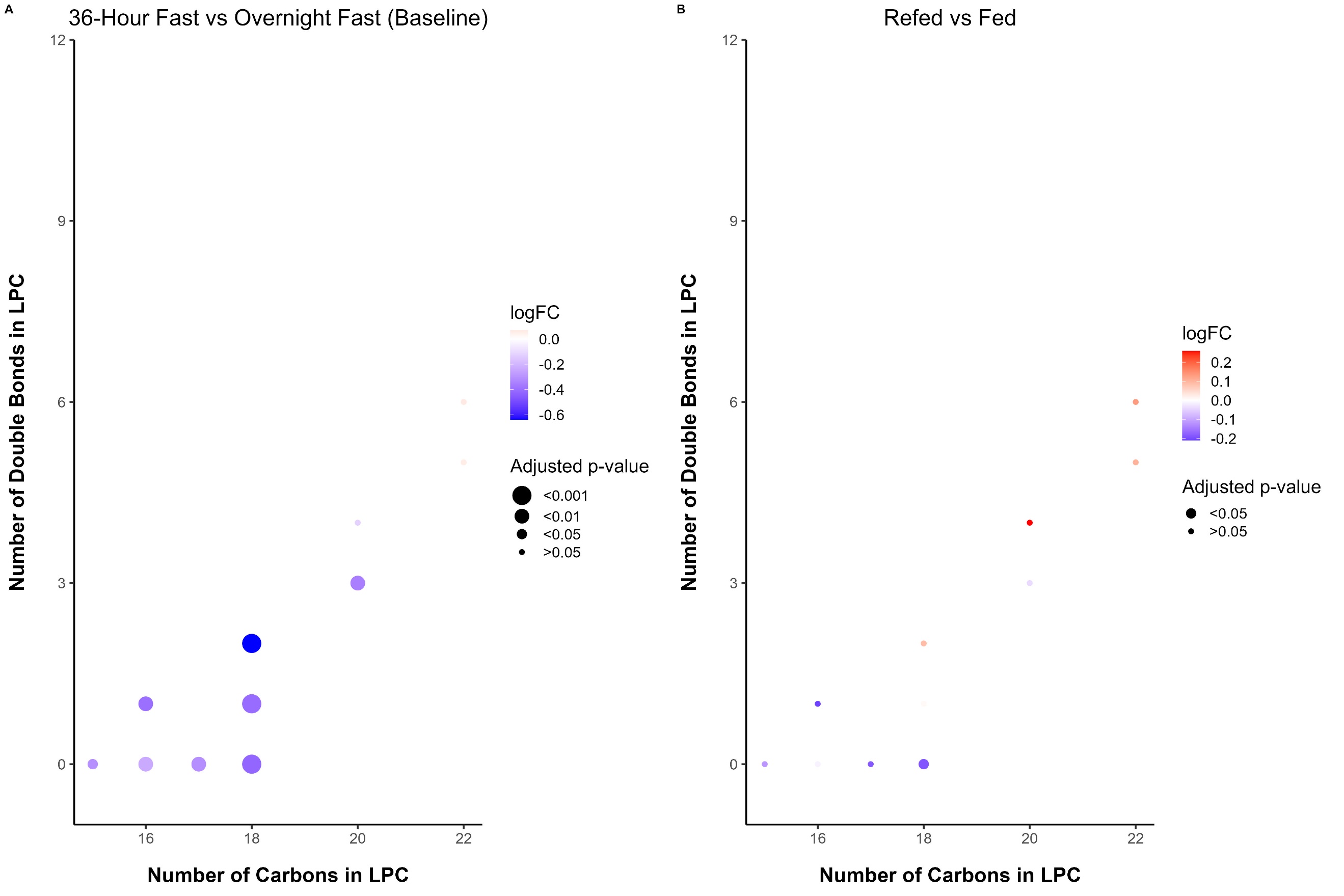


**Supplementary Figure S3.** Lysophosphatidylcholine (LPC) species comparing the A) 36-hour fasted state vs overnight fasted baseline, where higher logFC values indicate higher concentrations in the 36-hour fasted state, and B) LPC species comparing the refed state vs fed state, where higher logFC values indicate higher concentrations in the refed state.


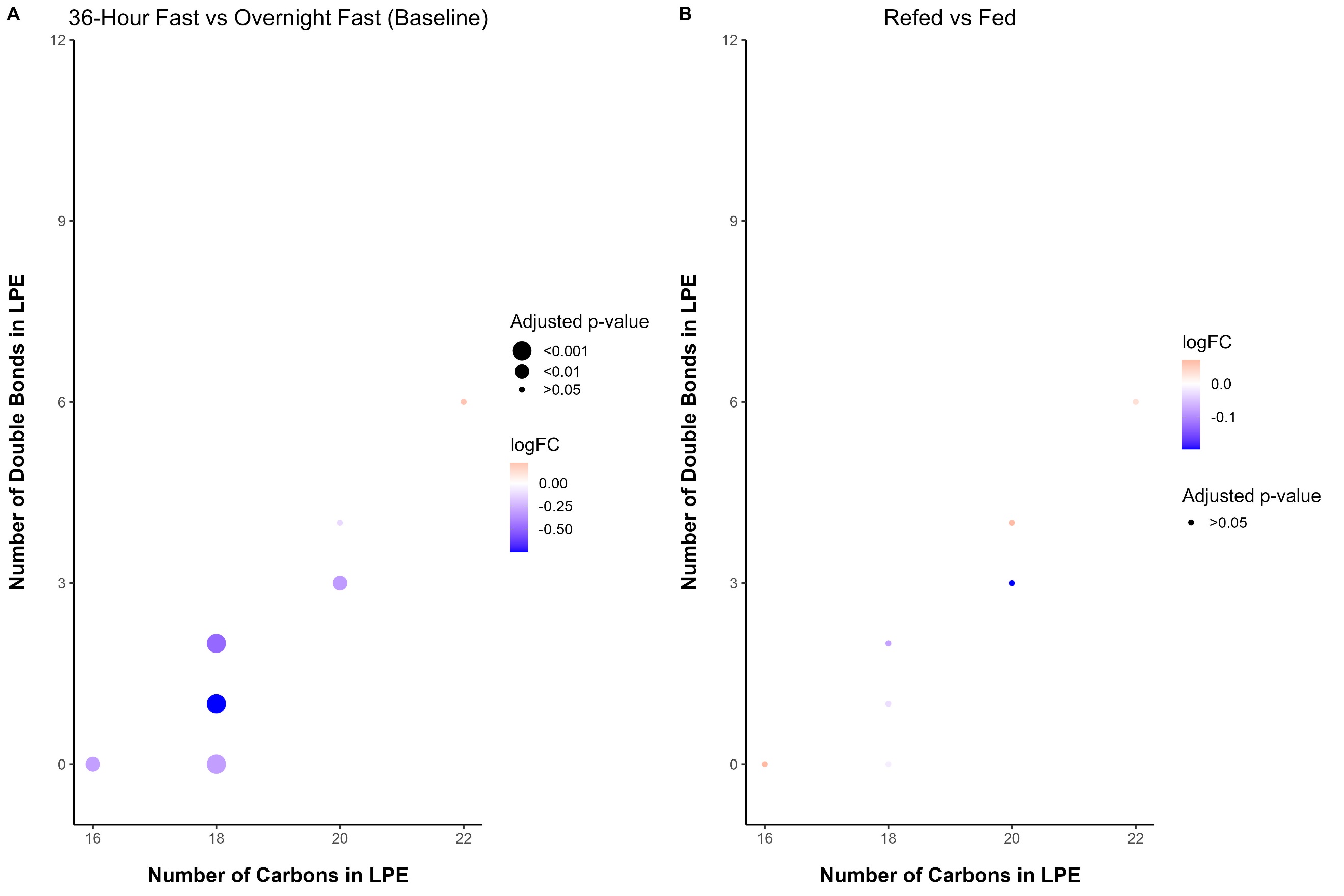


**Supplementary Figure S4.** Lysophosphatidylethanolamine (LPE) species comparing the A) 36-hour fasted state vs overnight fasted baseline, where higher logFC values indicate higher concentrations in the 36-hour fasted state, and B) LPE species comparing the refed state vs fed state, where higher logFC values indicate higher concentrations in the refed state.
